# Unlocking the Bottleneck in Forward Genetics Using Whole-Genome Sequencing and Identity by Descent to Isolate Causative Mutations

**DOI:** 10.1371/journal.pgen.1003219

**Published:** 2013-01-31

**Authors:** Katherine R. Bull, Andrew J. Rimmer, Owen M. Siggs, Lisa A. Miosge, Carla M. Roots, Anselm Enders, Edward M. Bertram, Tanya L. Crockford, Belinda Whittle, Paul K. Potter, Michelle M. Simon, Ann-Marie Mallon, Steve D. M. Brown, Bruce Beutler, Christopher C. Goodnow, Gerton Lunter, Richard J. Cornall

**Affiliations:** 1 Nuffield Department of Medicine and Wellcome Trust Centre for Human Genetics, Oxford University, Oxford, United Kingdom; 2 MRC Human Immunology Unit, Weatherall Institute of Molecular Medicine, Oxford, United Kingdom; 3 Department of Immunology, The John Curtin School of Medical Research, The Australian National University, Canberra, Australia; 4 Australian Phenomics Facility, The John Curtin School of Medical Research, The Australian National University, Canberra, Australia; 5 MRC Mammalian Genetics Unit, Harwell, United Kingdom; 6 UT Southwestern Medical Center, Dallas, Texas, United States of America; The Walter and Eliza Hall Institute of Medical Research, Australia

## Abstract

Forward genetics screens with *N*-ethyl-*N*-nitrosourea (ENU) provide a powerful way to illuminate gene function and generate mouse models of human disease; however, the identification of causative mutations remains a limiting step. Current strategies depend on conventional mapping, so the propagation of affected mice requires non-lethal screens; accurate tracking of phenotypes through pedigrees is complex and uncertain; out-crossing can introduce unexpected modifiers; and Sanger sequencing of candidate genes is inefficient. Here we show how these problems can be efficiently overcome using whole-genome sequencing (WGS) to detect the ENU mutations and then identify regions that are identical by descent (IBD) in multiple affected mice. In this strategy, we use a modification of the Lander-Green algorithm to isolate causative recessive and dominant mutations, even at low coverage, on a pure strain background. Analysis of the IBD regions also allows us to calculate the ENU mutation rate (1.54 mutations per Mb) and to model future strategies for genetic screens in mice. The introduction of this approach will accelerate the discovery of causal variants, permit broader and more informative lethal screens to be used, reduce animal costs, and herald a new era for ENU mutagenesis.

## Author Summary

Damaging mutations in single genes are an important source of information about the causes of disease, including more complex genetic disease; but these single gene disorders are typically rare in humans. An important strategy for identifying new disease mechanisms is to introduce multiple random mutations in mice and test the mice for biological differences; these mice act as models of human disease. However, discovering the disease-causing mutation is time-consuming and complex, requiring further generations of breeding. In this study we demonstrate a method that overcomes these problems by sequencing the entire genomes of multiple mice that have inherited a disease-causing mutation from a common ancestor. We use an algorithm that uses knowledge of all the mutations carried by the sequenced mice to identify the regions of the genome and mutations that are common to all the mice. Using this method we can rapidly link biological traits to genetic mutations. In contrast to current approaches, our strategy does not require large amounts of breeding, and it permits more accurate measurement of a wider range of traits; consequently its introduction will significantly reduce the number of mice required in the future, increase the number of traits that can be detected, and accelerate the discovery of new pathways and gene functions relevant to human diseases.

## Introduction

Forward genetic screens in mice carrying mutations introduced by the alkylating agent ENU can provide important and entirely novel insights into gene function [1], [2], [3], [4]. This approach does not require any prior assumption about mechanism, and by inducing random point mutations ENU generates viable phenotypes that mimic human disease. In the classic approach mice treated with ENU are bred to generate pedigrees segregating thousands of mutations, which are screened for phenotypes of interest. However determining which of the many induced mutations underlies the phenotype is a significant bottleneck in the process, requiring additional generations of breeding and outcrossing to another inbred laboratory strain in order to generate a linkage map, followed by sequencing of candidate genes or regions. This process is time consuming and costly. For example conventional fine mapping to obtain a linkage region of around 3 Mb (20–30 genes) requires at least 2 generations of additional breeding and genotyping 100–200 markers in 30–60 F2 mice. The need to propagate the mice tends to require non-lethal screens, which limits the range of assays and the scope to detect phenotypes. Furthermore, outcrossing can introduce unseen confounding variants affecting the trait, and tracking the phenotype through additional generations is complicated and can be unreliable [5].

Although whole genome and exome sequencing offer the prospect of accelerating discovery, current strategies remain dependent on conventional mapping [6], [7], [8], [9]. In this study we address the ENU bottleneck by showing how it is possible to use WGS and identity by descent to isolate a causative mutation rapidly and efficiently on a pure, single strain background, without the need for outcrossing or additional breeding. The strategy will allow the use of lethal and more informative screens. It will accelerate the discovery of new variants, permit a greater focus on novel mutations, and make forward genetics more accessible by reducing costs and broadening the screens.

## Results

### A Novel Strategy to Identify ENU Mutations

**Figure 1:**
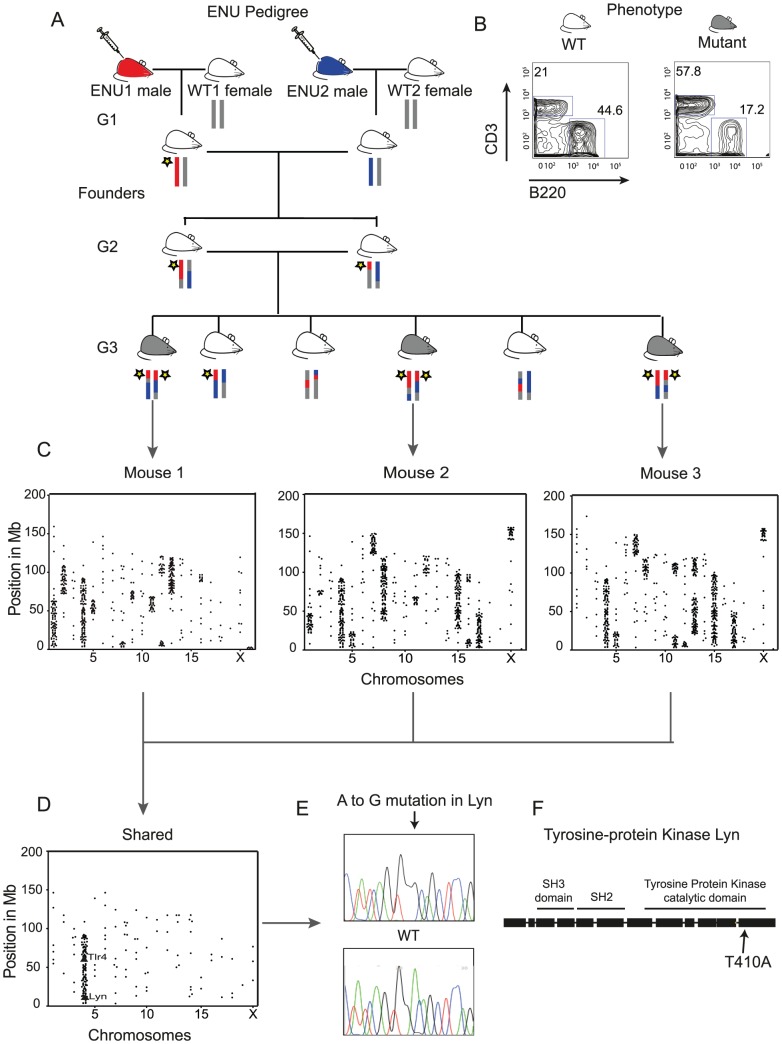
Whole-Genome Sequencing Identifies the IBD Homozygous Region and Causative ENU Mutation. (A) The structure of an ENU pedigree: two ENU treated males paired with WT B6 females generate founder G1 mice for the *ENU16CH17a* pedigree, and G3 mice exhibiting the phenotype are selected for WGS. Thus mice within the pedigree carry 4 possible haplotypes, ENU1, ENU2, WT1 and WT2. A yellow star illustrates the segregation of a causative variant. (B) Mice homozygous for the mutation exhibit B cell lymphopenia (here gating on blood lymphocytes). (C) Plots of homozygous filtered variants show the haplotype blocks across the chromosomes of each sequenced mouse. (D) Shared homozygous variants seen in all 3 sequenced mice cluster in an IBD region on Chromosome 4, containing exonic mutations in two genes, *Lyn* and *Tlr4*. (E) Confirmation of the *Lyn* A to G transition by Sanger sequencing. (F) The mutation lies in exon 12 within the catalytic domain.

In a typical strategy for generating and screening ENU mutant mice, C57BL/6J (B6) ENU-treated founders are bred with B6 females to generate G1 founders, establishing pedigrees in which pairs of G2 mice produce G3 mice segregating recessive and dominant mutations (Figure 1A). To assess the utility of WGS in the analysis of such mice, we chose a pedigree identified as *ENU16CH17a* where the recessive phenotype was peripheral B cell lymphopenia (Figure 1B and Figure S1), and performed WGS on three affected G3 mice from a single G2 pair to high coverage (average 24× per individual).

**Figure 2:**
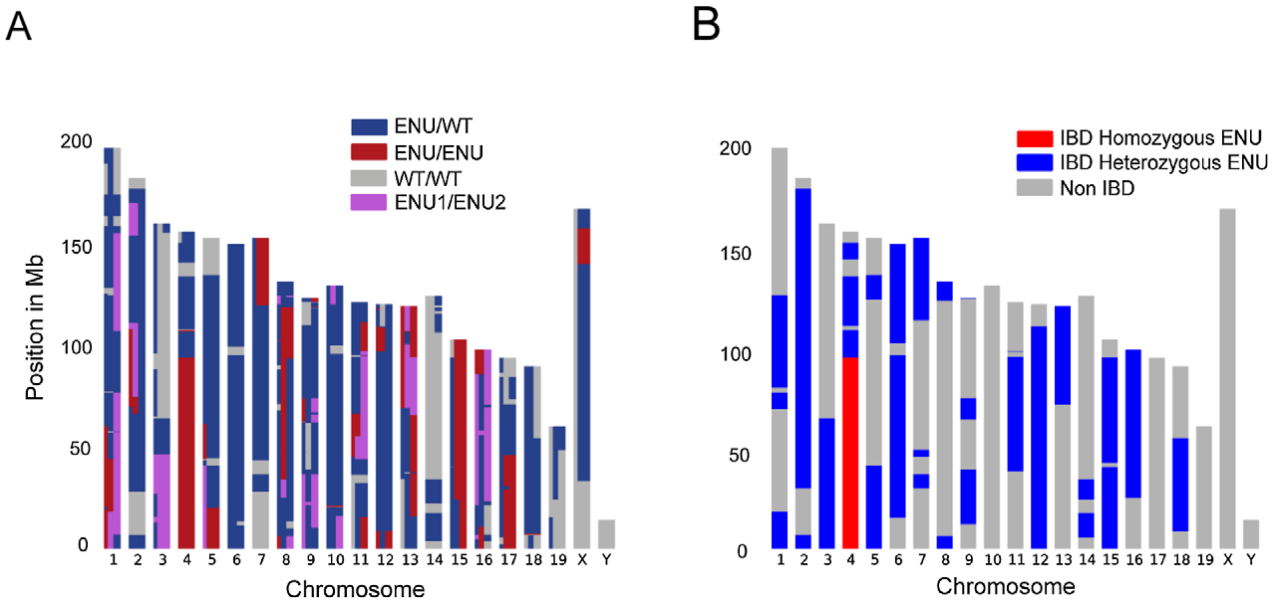
Identification of IBD Regions using a Modified Lander-Green Algorithm. (A) Graphical representation of the output of the algorithm, showing the genotypes for the 3 mice, based on combinations of the 4 haplotypes ENU1, ENU2, WT1 and WT2 inherited from the founder mice. WT1 and WT2 are genetically indistinguishable. Each mouse is represented by a vertical third of the plot for each chromosome, and color blocks represent unphased haplotype combinations for each mouse as indicated in the figure. ENU/ENU indicates homozygous ENU regions and ENU/WT indicates heterozygous regions for ENU 1 or ENU 2. (B) Graphical representation of the chromosomal IBD regions, showing shared heterozygous (blue) and homozygous (red) IBD regions. Regions are only IBD if all mice share alleles from a particular ENU founder, ENU1 or ENU2. Non homozygous IBD regions in which all mice carry at least one matching ENU allele are considered IBD heterozygous.

The causative variant in *ENU16CH17a* will belong to a haplotype that is shared by all the sequenced mice and inherited from an ENU-treated ancestor. By constructing chromosomal maps of the homozygous variants in all three animals we could demonstrate clustering of mutations within haplotype blocks inherited from ENU-treated founders (Figure 1C and Figure 1D). This suggested that the identification of IBD regions in multiple mice would be an elegant and efficient approach for mapping and identifying causative mutations. We developed a method based on the Lander-Green algorithm which uses genetic markers, knowledge of the pedigree and recombination rates, to infer the flow of alleles through the genealogy [10]. We modified the algorithm to exploit the partial knowledge of the G1 founder genotypes. Our implementation uses probabilistic variant calls to identify haplotypes from the four founder mice (ENU1, ENU2, WT1 and WT2) across the genome of each G3 individual (Figure 2A and Materials and Methods). The rate of ENU induced mutation is low and must be distinguished from both WGS artifacts and background variation from the reference, so we developed a series of filters to exclude non-ENU variants (Materials and Methods and Figure S2).

The haplotypes assigned by our algorithm identify the IBD regions (Figure 2B). IBD homozygous regions comprise 95.3 Mb (3.6% of the genome), containing 137 variants, including only two mutations in coding regions, both on chromosome 4 (Figure 1D). One mutation at position 3,710,143 was an A to G mutation inducing a missense change in the Src-kinase encoding gene *Lyn* (Figure 1E). The mutation corresponds to a threonine to an alanine substitution at amino acid residue 410 in exon 12 within the highly conserved Src activation loop in the protein kinase domain (Figure 1F). The phenotype seen in *ENU16CH17a* has been described in mice carrying a threonine to lysine substitution at the same codon in *Lyn* [11], indicating that the 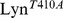 mutation is causative. The other mutation, at position 66,590,107, encoded a missense mutation in a single transcript of Toll-like receptor 4 reported by Ensembl (Tlr4-004 ENSMUST00000107365), but absent from the RefSeq dataset. *Tlr4* deficient mice do not have obvious defects in B cell development [12].

### Dominant Mutations

The *ENU16CH17a* pedigree carries a recessive functional mutation; however, to demonstrate the wider application of our method for dominant traits, we examined shared IBD heterozygous ENU mutations in the same G3 mice. The 3 *ENU16CH17a* mice share one or more haplotypes from a common ENU founder across regions comprising 40.8% of the genome (1083.8 Mb) (Figure 2B), containing 26 heterozygous candidate mutations shared by all 3 mice, comprising 25 missense and one splicing mutation; there were no nonsense mutations. PolyPhen-2 [13] predicted 9 as benign, leaving 17 heterozygous shared mutations with possibly deleterious effects. Sanger sequencing confirmed the presence of all 28 homozygous and heterozygous IBD variants (Table S1 and Table S2).

### The Frequency and Characteristics of ENU Mutations

The frequency of ENU mutations is dose related [14], [15] and may differ according to the mouse strain [16]. However, previous estimates of the ENU mutation frequency, which have ranged from 0.5 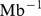 to 10 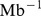, have been confounded by small datasets and locus specific bias [17], [18], [19], [20], [21], [22], [23].

**Figure 3:**
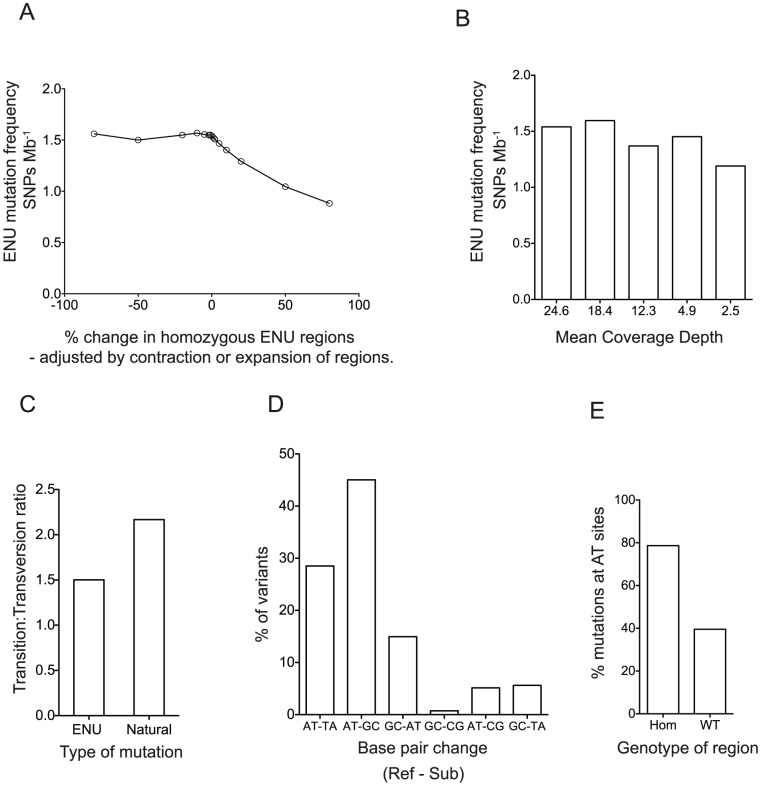
Characterization of the ENU Mutations. (A) The effect of expanding or contracting the homozygous ENU regions on the estimate of the ENU mutation frequency. (B) The effect of simulated depth of coverage on the estimated ENU mutation frequency. (C) Transition transversion ratio in homozygous ENU variants compared to a large dataset of non-mutagen induced laboratory mouse variation from the Centre for Genome Dynamics Mouse SNP Database. (D) The distribution of ENU mutations, showing reference base pairs and substitutions (ref-sub). (E) The proportion of homozygous mutations that occur at AT sites in homozygous ENU and homozygous WT regions. In each graph, columns or points show mean values across the 3 sequenced *ENU16CH17a* mice.

By observing the frequency of variants in the homozygous ENU regions and subtracting the background rate observed in homozygous WT regions, we have calculated the ENU mutation rate in the *ENU16CH17a* pedigree to be 1.54 mutations 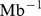. We excluded errors due to assignment of homozygous regions or inadequate coverage by modeling the effect of expansion or contraction of regions and of reduction in coverage (Figure 3A and 3B). The estimate of mutation frequency was also insensitive to changes in the assumed mutation frequency used in the algorithm to predict IBD regions - with assumed mutation frequencies in the range 0.25 to 3.0 mutations 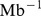, the estimated ENU frequency remained between 1.52 and 1.58 mutations 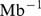 (Figure S3A).

Within the homozygous ENU regions, we could confirm the well-described transition∶transversion ratio and A-T base preference of ENU induced mutations [24], [25]. We found a 1.50∶1 transition: transversion ratio in ENU mutations compared to a 2.17∶1 ratio in naturally occurring mouse SNPs (Figure 3C). We confirmed the distinctive base preference signature of ENU mutations, which is mainly due to error-prone repair of 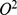 and 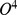 ethylthymidine leading to AT to TA transversions (28.5% of our mutations), and AT to GC transitions (45.0% of our mutations), respectively [26], [27] (Figure 3D). We found 78.7% of all homozygous mutations were at A:T sites, compared to the 58% AT content of the mouse genome [28] and different to non-ENU variants seen in homozygous WT regions, of which 39.5% were at AT sites (Figure 3E).

### Identification of IBD Regions and Mutations at Low Coverage

**Figure 4:**
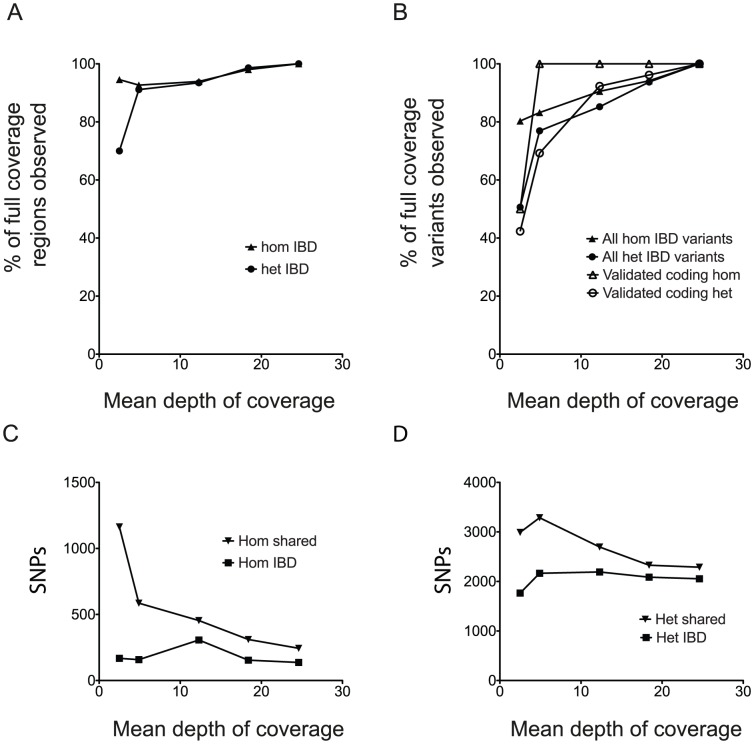
The Effect of Reduced Coverage on the Assignment of Regions and Variants by IBD. (A) The proportion of ENU homozygous and heterozygous IBD regions from the full 24× coverage dataset identified at simulated lower depths of coverage per mouse. (B) The proportion of homozygous and heterozygous IBD variants from the 24× coverage dataset identified at simulated lower depths of coverage per mouse. The validated variants are the coding or splice variants confirmed by Sanger sequencing (Table S1). (C) The number of IBD homozygous SNPs at different simulated coverage depths compared to the number of shared homozygous SNPs across all 3 mice. (D) The number of IBD heterozygous SNPs at different simulated coverage depths compared to the number of shared heterozygous SNPs across all 3 mice.

For a high throughput ENU program, an analysis based on IBD would ideally identify SNPs and IBD regions accurately even at low coverage per individual. Therefore to model this we simulated a lower coverage dataset by randomly selecting subsets of *ENU16CH17a* reads and checked the consistency of variant calls at different simulated levels of coverage compared to 24× per mouse. The assignment of IBD regions remained highly consistent with the complete dataset down to very low coverage levels (Figure 4A and Figure S4). At 5× coverage per mouse, 93% of homozygous and 91% of heterozygous IBD regions seen at 24× were assigned, and 83% of homozygous and 77% of heterozygous variants in IBD regions overlapped with those found in IBD regions at full coverage (Figure 4B). Within the validated set of non-synonymous coding and splice site mutations, we identified all the homozygous IBD variants (2/2), and 69% of heterozygous IBD variants (18/26) at 5× (Figure 4B).

We compared our IBD approach to a simple method of selecting all variants observed in all 3 affected mice. At lower coverage levels the simple approach identified very large numbers of homozygous shared variants compared to the IBD method; at 5× coverage there were 586 shared variants compared to 158 by IBD (Figure 4C). This error is likely to be due to the miscalling of heterozygous variants as homozygous, coupled with accumulation of further heterozygous variants, since the overall number of heterozygous shared variants at each simulated depth is greater than that observed using IBD (Figure 4D). By only considering variants in IBD genomic intervals, we distinguished homozygous from heterozygous variation more accurately and reduced the number of spurious shared variants, making isolation of causative mutations feasible at low coverage.

To confirm the utility of our method at low coverage experimentally, we Sanger sequenced shared variants from 3 affected mice in a second ENU pedigree, which had been sequenced by WGS at 4× per mouse. We again identified the causative recessive mutation, and found true-positive rates of 85.7% (24/28) for homozygous variants and 86% (48/56) for heterozygous variants. Within the subset of coding variants, we identified 100% (4/4) of the homozygous variants, including the causative variant, and 96% (24/25) of the heterozygous variants (Table S4). As expected, Sanger sequencing of variants from non-IBD regions revealed lower true-positive rates, 57% (49/86) of called variants were confirmed by Sanger sequencing, comprising 59% (10/17) of coding variants and 57% (39/69) of non-coding variants (Table S5), demonstrating once again the greater accuracy of variant calling in IBD regions compared to non-IBD regions at 4× coverage per mouse.

### Modeling the Efficient Generation of ENU Libraries

To explore how our computationally efficient and rapid route from phenotypes to candidate genes could be applied to a large-scale ENU program, we proceeded to model the inheritance of mutations within a typical ENU pedigree.

**Figure 5:**
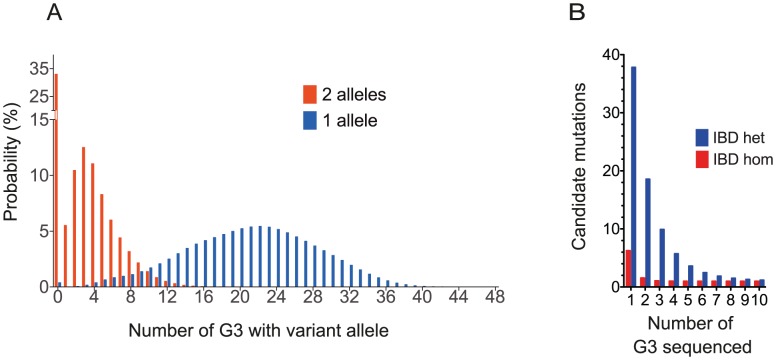
Modeling the Frequency of Mutants and the Power to Assign Causation by WGS. (A) The frequency distribution for all mutations within a pedigree at the G3 level, based on a model pedigree of 48 G3 arising from 4 G2 pairs (Materials and Methods). In the specific case of mutations causing fully penetrant phenotypes, the histograms show the distribution of affected mice with recessive (2 allele) and dominant (1 allele) traits. (B) The number of IBD candidate mutations, defined as missense, stop or splice-variants, as a function of the number of sequenced affected G3 mice, based on our model.

First we asked how frequently a hypothetical fully penetrant ENU mutation causing a screened phenotype would be observed among the G3 mice in the proposed breeding strategy. We assumed that a G1 pair could give rise to 4 stable G2 pairs, each generating 3 litters with a conservative estimate of 4 live mice per litter. A single pedigree would then generate 48 G3s for screening (Figure 1A). Using a probabilistic model incorporating all the possible inheritance patterns (Materials and Methods), we calculated that 51% of ENU mutations present in the founder mice occur 3 times or more as homozygous within the set of G3 mice, and 62% occur twice or more. 99.6% of mutations would be present at least once as a single allele in the 48 G3 mice, 98% at least 6 times and 95% at least 9 times (Figure 5A).

Next, we asked whether, under the proposed strategy, the number of non-causative candidate mutations would be sufficiently small to efficiently exclude these mutations. The number of candidate mutations IBD in affected G3 mice from a single pedigree can be estimated as a function of the number of affected G3s sequenced, the empirical ENU mutation frequency of 1.54 mutations 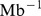 (Figure 3B), the relatedness of the mice and the proportion of mutations that affect protein sense. We found on average 1.4% (9.3/647.3) homozygous mutations in each G3 mouse lie in exons or splice sites; 73% of this subset caused missense or splice site mutations; we identified no homozygous stop mutations in *ENU16CH17a*. Thus we derive that 1.05% of mutations affect protein sense. Using these parameters we calculate that sequencing 3 or 6 mice will reduce the number of candidates to 1 homozygote or 2–3 heterozygote mutations, respectively (Figure 5B and Materials and Methods). This model is consistent with the empirical data from the *ENU16CH17a* pedigree.

## Discussion

The importance of this study is in showing how low coverage WGS of multiple mice with a phenotype can identify causative ENU mutations without the need for out-crossing, or knowledge of dominant or recessive inheritance. This strategy simultaneously generates linkage maps and identifies the shared mutations with a high degree of confidence. The advantage of our linkage-based approach is that ENU-induced mutations from multiple affected mice can be used to track the IBD regions and then isolate the causative mutation.

Linkage analysis using exome sequencing has been effectively harnessed for the study of human pedigrees. Variations on the Lander-Green method, developed for array data, incorporate knowledge of the population allele frequencies of HapMap SNPs [29], [30], [31]. Alternative approaches use other hidden Markov models (HMM) to identify regions that are IBD and common to autosomal recessive phenotypes [32], [33]. Reducing the search space from the whole genome to the exome significantly reduces the number of informative variants [34], however this is typically several orders of magnitude larger than the number of ENU-induced exonic variants, e.g. 6000 to 8000 exonic HapMap variants per individual [29], [30] compared to 74 exonic variants in our ENU pedigree. The low frequency of ENU coding variants does not permit fine scale linkage analysis based only on exonic variants. Exome sequencing also precludes the detection of regulatory mutations [35], and the inefficiencies of capture have resulted in a failure to find the causative mutant in one in five ENU pedigrees, even for recessive traits [8], [36]. The ability to detect IBD regions using low coverage and the falling costs of next generation sequencing make our WGS method increasingly cost effective.

Knowledge of the ENU mutation frequency allows us to model an efficient sequencing strategy. Our data show that sequencing 3 affected G3 mice with a recessive trait or 6 mice with a dominant trait would yield on average 1 or 2 candidate IBD mutations. The Lander-Green algorithm on which our IBD analysis is based, scales exponentially with the number of individuals in the pedigree, but remains computationally feasible with a pedigree of 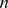 individuals and 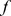 founders where 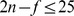 [31], [37]. The algorithm would accommodate further refinement to take into account the known characteristics of ENU mutations (Figure 3D). By generating haplotype data for many ENU pedigrees, our approach will also eventually lead to a fine scale map of active recombination sites in the mouse, which, unlike existing maps based on recombinations that arose historically between outbred strains of mice [38] or more recently between intercrossed inbred strains [39], is unbiased by selection or strain differences. Such a map could then be used to optimize the performance of our Lander-Green based algorithm.

We believe that the adoption of our approach by large-scale ENU programs will lead to a substantial increase in the productivity of the programs, advancing our understanding of gene-function and the mechanisms of genetic disease. Our approach will reduce the burden in animal costs and allow post-mortem screens, with increased sophistication and accuracy in a broader range of tissues. With the rapidly falling costs of WGS we can envisage a future in which all G3 ENU mice are sequenced to a depth sufficient to identify and segregate all their mutations, creating a rich dataset of allelic variation and corresponding phenotypic information, including linkage data for non-coding mutations with measurable effects. This could be achieved accurately with 4–5× sequencing due to the increased power to impute genotypes. This database could be mined for associations across pedigrees, including the detection of subtle phenotypes.

## Materials and Methods

### ENU

*ENU16CH17a*ENU treated B6 mice were generated and screened at the Australian Phenomics Facility, The Australian National University, Canberra [40]. Male founder mice for each pedigree, 8–15 weeks old, were treated three times 1 week apart with 90–100 mg/kg *N*-ethyl-*N*-nitrosourea (Sigma) prepared in 10% ethanol, citrate buffer (pH 5.0). After 8 weeks, the treated mice were mated with B6 females. Individual G1 progeny were intercrossed to generated G2 pairs. Phenotypic screening of G3 mice included flow cytometry of peripheral lymphoid cells (Figure 1A). We obtained tail DNA from 3 affected G3 siblings from the *ENU16CH17a* pedigree.

### Whole-Genome Sequencing, Mapping, and Variant Calling

We performed DNA sequencing on an Illumina HiSeq 2000 machine, using two lanes per mouse. 100 bp paired-end reads were generated. We mapped reads to the mouse reference genome MGSCv37 using Stampy [41] with BWA settings. 94.5% of genome was covered at least once, mean coverage across the genome 24 fold per mouse. An in-house variant caller Platypus was used, version 0.1.8 (www.well.ox.ac.uk/platypus A.J.R, Mathieson I, G.L, McVean G, (2012)). We annotated the variants using Annovar [42] with Ensembl (release 64) gene annotation. Functional predictions were made using Polyphen-2 using probabilistic classifications based on a model trained with the HumVar dataset, tailored for detection of Mendelian disease caused by mutations with large effects. Although the training dataset consists of human disease causing mutations, the modeling is based on sequence and structural features applicable across species [13] and higher Polyphen-2 scoring has been shown to correlate with damaging murine ENU mutations [36]. The 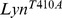 mutation in *ENU16CH17a* was confirmed independently using exome sequencing [36].

### Variant Filters and Union File

We developed a pipeline to filter the variant calls. As a first step we eliminated variants previously observed in two or more other ENU pedigrees using a variant union file (Figure S2), since these could be assumed to be due to systematic error, for example in repetitive regions with mismapping, or true non-ENU variation from the reference mouse sequence. To create this union file of shared, and thus non-ENU, variation, Platypus was used to call variants from mice from 9 different ENU pedigrees simultaneously. Thus at each variant locus a genotype and genotype likelihood was assigned for all mice. The nine pedigrees included 6 from the Australian Phenomics Facility at the Australian National University, 2 from the MRC Harwell Centre for Mouse Genetics and one from the Beutler Group at The Scripps Research Institute. The Harwell pedigrees and one of the ANU mice were on a mixed strain background. All other mice were on a straight B6 background (as was *ENU16CH17a*). We identified 7,624,313 unfiltered calls amongst these 9 pedigrees from 3 centers (Figure S2A). In order to exclude ENU variation we removed variants observed in only one pedigree. 83.6% (6,371,574/7,624,313) of raw variant calls were shared by more than one pedigree (Figure S2B). Within this dataset of shared variants there were 6,371,548 unique genomic positions, 81.7% were SNPs (5,203,298) and 18.3% (1,168,250) indels). transition∶transversion ratio among SNPs was 1.9% (3,394,771 transitions, 1,805,983 transversions, 2,544 have more than 2 alleles). To examine the proportion of this shared variation attributable to B6 reference strain mice, we excluded shared variants exclusively observed in mixed strain mice. The resultant shared variants are observed in at least 2 pedigrees, including at least one fully B6 pedigree. Since all pedigrees included from MRC Harwell are mixed strain, the MRC Harwell variants fully overlap the other laboratories (Figure S2C). The large majority (91.8%) of non-ENU variation in B6 mice is not laboratory specific, suggesting isolated genetic drift within individual colonies accounts for little of the observed variation. 24.1% of the shared dataset were present in dbSNP.

After filtering non-ENU variation from the *ENU16CH17a* calls, we removed any additional known variants from dbSNP (version 128) and filtered out remaining calls that were clustered closely together with a threshold of less than 1,000 bp. The variants were then filtered for Phred based quality score assigned by the variant caller 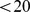, removal of calls that failed Platypus allele bias or strand bias filters, removal of variants with a high local frequency of bad reads, filters for homopolymers and repetitive sequence, including di-nucleotide repeats of 20 bp or more. Indels were removed as ENU overwhelmingly causes point mutations. Finally loci with coverage in the upper or lower 1% of the coverage distribution were excluded. All coverage distributions were measured with BEDTools [43].

### Lander-Green Based Algorithm

The Lander-Green algorithm [10] uses a combination of genotype information from informative markers (here, the SNP genotypes called from the next-generation sequencing data), and knowledge of local recombination rates to determine the ancestral haplotypes in specific genomic intervals, and the locations at which recombinations and therefore transitions between inheritance state vectors occur.

The pedigree used in this experiment (Figure 1A) can be considered to originate with the G1 pair of common ancestors who each carry a combination of the haplotypes inherited from the G0 mice, these being ENU1, ENU2, WT1 and WT2. Thus for the purpose of the algorithm the pedigree consists of 5 non-originals – that is 5 individual mice with parents in the pedigree.

The Lander-Green algorithm represents the ancestral haplotypes of the 5 non-originals as a state vector with 10 binary co-ordinates, representing the 5 individual mice, arising from 10 gametes. Within each inheritance vector a 0 coordinate indicates a gamete carrying grand-paternal DNA at a locus, and 1 indicates grand-maternal inheritance. These are arbitrarily phased. There are 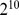 possible state vectors.

The standard HMM machinery is well documented [44]. A HMM has two main components which are model-specific: these are the state transition matrix, which specifies the probabilities of transitions between any two model states, and the likelihood, which is the probability of the observed data given a particular model state. The Lander-Green algorithm uses a state transition matrix based on the recombination rate, which encodes the probabilities of transitions between any of the ancestral state vectors, based on the number of recombinations required for the transition. In our implementation we use a recombination map [39] to compute average local recombination rates across the genome. We do not store the whole transition matrix in memory, but compute matrix elements on demand. This is straight-forward, as all matrix entries can be expressed as powers of the recombination rate and one minus the recombination rate. This vastly reduces the memory requirements for the algorithm, which are now linear in the number of state vectors rather than quadratic.

For each G3, a state vector determines which two ancestral haplotypes that make up G1, make up the local genotype, e.g. (ENU1, WT2). We compute a probability for the observed SNP genotypes, given each ancestral state vector, in 100 kb windows across each chromosome. This probability has two components: a prior probability of observing a SNP in the given window, and genotype likelihoods computed by the variant caller from the sequence data. We assume fixed priors of observing a SNP in ENU haplotypes (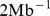) and WT haplotypes (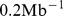). The IBD regions inferred by the algorithm were relatively insensitive to changes in these priors (Figure S3B).

The likelihood for a particular state vector is the sum over all possible combinations of the SNP genotypes (0/0, 1/0, 1/1) for the 3 mice, of the product of the SNP priors and the relevant genotype likelihoods for the 3 G3 mice. This incorporates the dependent relationships between the mice. In the case of multiple SNPs occurring in the same window, we assume that the SNPs are independent, and the likelihood for all mice in the window is the product of the likelihoods across all SNPs. Using the genotype likelihoods from the caller allows us to accommodate errors in the WGS data; a modification to the conventional Lander-Green algorithm that has been used to infer IBD in array data. [45].

Due to the paucity of polymorphic sites in the in-bred B6 mouse, there are many 100 kb windows which contain no SNPs. If no SNPs were called in a window, the most likely explanation is that no SNPs were present. In a small fraction of cases, a real SNP will be missed due to low coverage or variant-calling errors. To deal with windows that contain no SNPs, we supply a set of likelihoods weighted towards 0/0. Specifically we assume a 1/10 probability that a heterozygous SNP was missed, and a 1/100 probability that a homozygous SNP was missed.

Finally, we use the forward backward algorithm [44] to compute the posterior probability of each state vector at each window. We select the state vector with the highest posterior in each window to construct the sequence of most probable inheritance states across each chromosome. This information is shown in Figure 2A.

The program was coded in Python (http://www.python.org) and Cython (http://cython.org). The code is freely downloadable from http://www.well.ox.ac.uk/lgenu. Graphical plots of genotypes were generated using matplotlib (http://matplotlib.sourceforge.net).

Within our three generation pedigree, frequent recombinations, particularly from state A to state B and back to state A within a small genomic interval are likely to be artifactual. Therefore we performed a smoothing step on the output inheritance states, such that recombinations from state A to state B and back to state A within 1 Mb, within an allele, are corrected to state A.

In reporting the results we define IBD heterozygous throughout to refer to regions or variant sets in which all sequenced individuals share at least one allele from the same ENU founder but are not IBD homozygous.

### Frequency and Characteristics of ENU Mutations

Our dataset includes on average 647 mutations per mouse in homozygous regions spanning over 1,000 Mb across 3 mice (mean 405 Mb, 15.3% of the genome, per mouse). Sanger sequencing of the candidate mutations confirms the reliability of our filtered call set (Table S1). By observing the frequency of these mutations and subtracting the low background frequency from homozygous WT regions, we estimated the ENU mutation frequency, and used the homozygous ENU region variants to examine the base preference of ENU mutations. Comparison of the transition transversion ratio was made with all the variants in the Centre for Genome Dynamics Mouse SNP Database (http://cgd.jax.org/cgdsnpdb) on 8.5.12. This database includes over 66 million SNPs from 136 inbred laboratory mouse strains predominantly imputed from the mouse diversity array [46], and is representative of the characteristics of naturally occurring (non-ENU) SNPs in an inbred mouse.

### Simulating Lower Coverage Depths in Our Empirical Dataset

We generated random subsets of reads from the *ENU16CH17a* bam file using a script that utilizes pysam, a SAMtools [47] interface for Python. For details see http://code.google.com/p/pysam/. We called and filtered variants on the down-sampled bam files using the same pipeline described above. Comparisons of IBD regions and variant calls between the lower coverage datasets and the full (24×) coverage data were made using BEDTools [43] to measure intersections. The intersections of IBD regions were analyzed by a per base pair comparison.

### Comparison with Non–IBD Approach to Detect Shared Variation

To examine whether an IBD method performs better than a simple per SNP approach to detect shared variation in the 3 mice attributable to ENU we generated a comparison set of shared variants at each simulated coverage depth by selecting variants that were observed in all 3 mice. In exactly the same way as with the IBD variants we included SNPs in which there was at least one homozygous or heterozygous mouse and the remaining 0, 1 or 2 mice had no genotype information (denoted 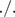 in the vcf file) or a reference (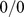) genotype call with at least one variant call and less than 5 supporting reference reads.

### Calculating the Proportion of Mutations Affecting Protein Sense

To examine the distribution between missense and splice mutations we looked at the larger dataset of heterozygous mutations. Across our three mice, 86% (21.7/25) of potentially damaging mutations were missense mutations, 6% (1.3/25) were nonsense mutations and 8% (2/25) were in splice sites. A large database (http://mutagenetix.utsouthwestern.edu/ on 8.5.12) of over 5,000 incidental ENU mutations with no observed deleterious effects, identified in the course of next generation sequencing (Applied Biosystems SOLiD), reports 85.9% missense, 4.4% nonsense and 9.7% splicing mutations, and agrees broadly with our findings.

### Modeling Expected Numbers of ENU Mutations in G3 Mice

To model the segregation of mutations within a pedigree, we calculated the probability of a mutation being inherited by a G3 under the three possible situations where the G2 parents carry 0, 1 or 2 copies of the mutation (in 25%, 50% and 25% cases respectively; individual G2 mice are not homozygous for any ENU mutation). We denote the chance of inheriting the mutation at G3 under each of these situations with a given zygosity (homozygous or heterozygous) at G3 as 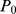, 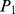, and 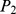. Clearly some of these probabilities will be 0.

Conditional on the number of mutations carried by the G2 parents, the number 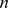 of G3 offspring inheriting the mutation with the required zygosity can be modeled using a binomial distribution. For a given G2 parent pair, we denote this distribution by 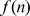. Assuming that each G2 pair produces 3 litters of 4 live mice, this distribution is given by
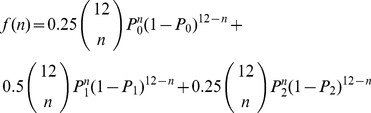
(1)Here 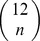 or 12 choose 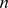 denotes the binomial co-efficient indexed by 12 and 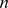. To estimate the probability of 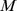 G3 carrying the mutation across the 48 G3 from 4 G2 pairs, we can convolute across all combinations of mice that together transmit precisely 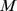 mutations with the required zygosity from G2 pairs, such that
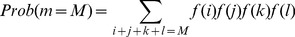
(2)We considered two situations: one of a recessive mutation, in which a G3 has two alleles from parents that are heterozygous for the mutant; and the situation of a dominant mutation, where we considered that homozygotes may also have the phenotype and may be indistinguishable from heterozygotes. In this way it is possible to calculate the probability of any recessive or dominant mutation carried by a founder occurring M times in the G3 mice. The results are presented in Figure 5A.

### Modeling Numbers of Shared IBD SNPs in Multiple Sequenced Mice

We modeled the proportion of the genome expected to be IBD in 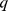 sequenced mice without accounting for linkage to the causative mutation. We calculated the probability of 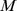 G3 carrying a shared ancestral haplotype at any locus as described above in equation 2 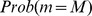, for each 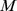 between 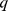 and 48 (the modeled number of G3 mice), and calculated a probability of picking 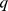 mice sharing such a locus by chance from a pool of 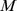 mice sharing the locus and 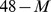 individuals not carrying the locus. Since each mouse can only be picked once this corresponds to a hypergeometric distribution. We then summed over the product of this and the 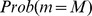 for each 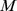 between 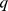 and 48 to get the overall probability, 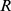, of any unlinked locus being observed in all the affected sequenced mice.
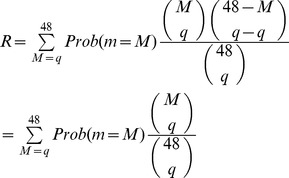
(3)

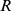
 is the proportion of the genome expected to be IBD for a specified number 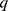 of sequenced mice. We used our knowledge of the ENU mutation rate (1.54 mutations 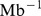), and the fraction of variation affecting protein sense (1.05% missense, nonsense or splicing), to estimate the number of homozygous or heterozygous candidate mutations shared by 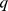 affected sequenced mice.
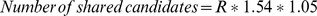
We add one mutation to model the causative mutation which is always present. In phenotypically affected mice a region from the ENU founder persists around the causative mutation due to linkage, and this adds another fraction 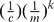 of the genome, where c is the fractional size of the chromosome, m is the number of meioses per G3 mouse, and k is the number of G3 mice. This approximates to a further 0.7 mutations or 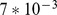 candidate coding mutations. Since this is negligible we simply approximate to 1 additional mutation. The results are presented in Figure 5B.

### Sanger Sequencing

We amplified the 28 candidate mutations with two rounds of PCR from genomic DNA using internal and external fully nested primers (Table S3) and then amplified with Big Dye (Applied Biosystems Ltd) before sequencing on an Applied Biosystems 3720xl machine. All nested sequencing reactions were run in duplicate to check for PCR error. We carried out Sanger sequencing to validate shared variants from the 3 mice sequenced at low coverage from a second pedigree using primers shown in Table S4 and Table S5.

### Statistical Methods

Analysis of means was performed using the Graphpad Prism 5 package. All other analyses were written in custom scripts and described in the Materials and Methods.

### Ethics Statement

All animal experiments were approved by local and national ethical review, including the Australian National University Animal Ethics and Experimentation Committee and the Oxford University Local Ethical Review Committee and UK Home office License PPL 30/2455.

## Supporting Information

Figure S1Splenic B Cell Populations in ENU16CH17a. (A) B220 and CD93 within the 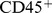 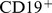 B cell population. (B) Immature B cells gated on 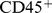 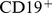 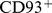, indicating the T1 (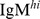 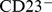), T2 (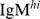 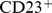) and T3 (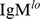 ) populations. (C) Mature B Cells gated on 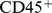 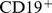 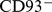. Follicular (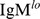 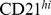) and marginal zones (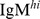 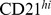). (D) Follicular B cells gated on 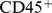 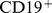 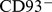 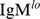 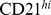 showing 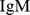 vs 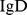, expression of 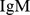 falls and 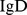 increases with maturity of follicular B cells.(EPS)

Figure S2 Variant Union File—Distribution of Variations by Laboratory. (A) All variants called over 9 ENU pedigrees from 3 centers. (B) All variants observed in more than one pedigree. (C) All variants seen in more than one pedigree including at least one pedigree on a straight B6 background. Each shape in the Euler diagrams represents the pedigrees from a single centre.
(EPS)

Figure S3Effect of Modeled ENU Mutation Frequency Input to Lander-Green Algorithm on Output Measures. (A) Effect of input ENU mutation frequency on the estimated ENU mutation frequency from the IBD regions. We ran the Lander-Green algorithm using a range of assumed ENU mutation frequencies as inputs, and estimated the mutation frequency from the data generated as described in Materials and Methods. (B) Effect of input ENU mutation frequency on the assigned IBD homozygous and heterozygous regions. All regions are compared to those generated using an input rate of 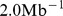. Loss of regions is % loss compared to the regions at 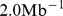. Gain is % of regions assigned using each input that were inconsistent with those seen at 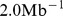 input.(EPS)

Figure S4 IBD Regions Assigned at each Simulated Coverage Level. Plots of IBD regions from the assigned inheritance states for each mouse, shown at different simulated coverage levels. Coverage level is the mean depth per mouse.
(TIF)

Table S1 Candidate Variants. The total shared homozygous or heterozygous regions contained 2,951 mutations from the filtered call set (2,191 were present in all 3 mice), including the 2 non-synonymous homozygous mutations, and 30 heterozygote missense, nonsense or putative splice site mutations; 28 candidate homozygous or heterozygous variants were present in all 3 mice.
(PDF)

Table S2Variants in IBD Regions with Inconsistent Genotypes. 4 protein sense changing variants within the IBD regions were rejected as causative because they were absent in one or more affected animal, despite good individual depth of coverage (threshold 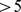 good quality reads at the locus for the inconsistently genotyped animal).(PDF)

Table S3 Forward and Reverse Primers used to Validate Candidate *ENU16CH17a* Variants.
(PDF)

Table S4 IBD Variants in the Second Pedigree Sequenced at Low Coverage (4× per individual) used to examine the false discovery rate at low coverage. The variants in this low coverage pedigree were a randomly selected subset of the filtered shared IBD variants and included both coding and non-coding mutations. Sanger sequencing results: True Positive (TP), False Positive (FP) Failed Sequencing (Unknown - assumed FP).
(PDF)

Table S5 *Non-IBD* Variants in the Second Pedigree Low Coverage. Non-IBD variants selected randomly from the low coverage pedigree (4× coverage per mouse), showing SNP genotype from variant caller for each mouse. Sanger sequencing results: True Positive (TP), False Positive (FP) Failed Sequencing (Unknown - assumed FP).
(PDF)
