# Supplementary figures and images for: Unlocking the Bottleneck in Forward Genetics Using Whole-Genome Sequencing and Identity by Descent to Isolate Causative Mutations
Source: PLoS Genet. 2013 Jan 31;9(1):e1003219. doi: 10.1371/journal.pgen.1003219 (PMC3561070; doi:10.1371/journal.pgen.1003219)

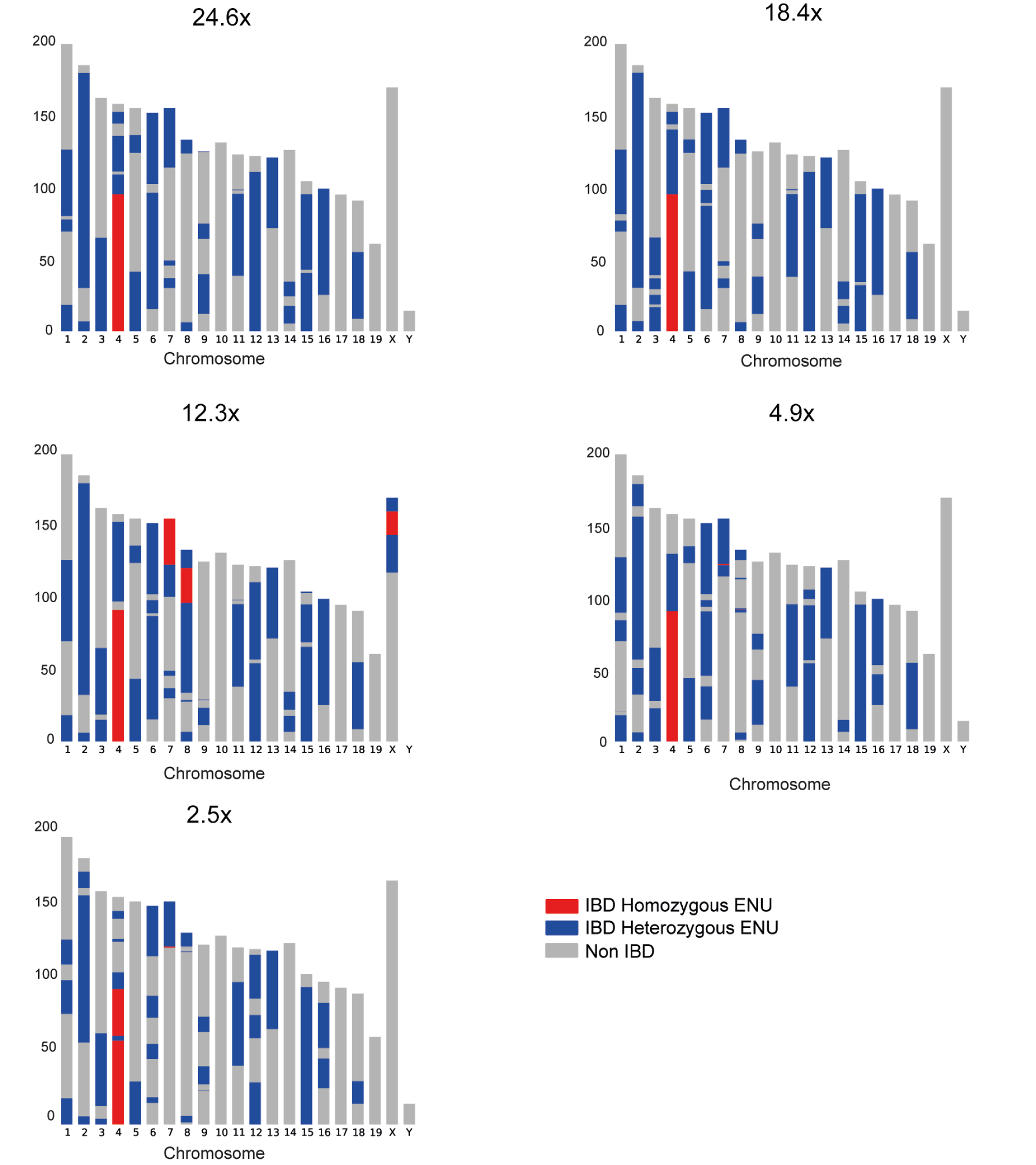

Supplement: Figure S4 — IBD Regions Assigned at each Simulated Coverage Level. Plots of IBD regions from the assigned inheritance states for each mouse, shown at different simulated coverage levels. Coverage level is the mean depth per mouse. (TIF) [file pgen.1003219.s004.tif]
