# Supplementary material for: Unlocking the Bottleneck in Forward Genetics Using Whole-Genome Sequencing and Identity by Descent to Isolate Causative Mutations
Source: PLoS Genet. 2013 Jan 31;9(1):e1003219. doi: 10.1371/journal.pgen.1003219 (PMC3561070; doi:10.1371/journal.pgen.1003219)
Supplement: Table S1 — Candidate Variants. The total shared homozygous or heterozygous regions contained 2,951 mutations from the filtered call set (2,191 were present in all 3 mice), including the 2 non-synonymous homozygous mutations, and 30 heterozygote missense, nonsense or putative splice site mutations; 28 candidate homozygous or heterozygous variants were present in all 3 mice. (PDF) [file pgen.1003219.s005.pdf]

Chr	Pos	Gene	Ref	Sub	AA sub	Exon	Sanger	Polyphen (Humanvar)	genotypes 1 2 3	type
2	120317562	Capn3	C	T	T341M	exon9	TP	0.996/ prob	0/1 0/1 0/1	nonsynon
2	148509797	Gzf1	G	A	A71T	exon3	TP	0.009/benign	0/1 0/1 0/1	nonsynon
2	148697743	Cst3	A	G	W126R	exon3	TP	0.564/ poss	0/1 0/1 0/1	nonsynon
2	164482725	Wfdc10	T	A	M85K	exon2	TP	0/ benign	1/0 1/0 1/0	nonsynon
3	53343180	Frem21	A	T	V76D	exon3	TP	0.621/poss	1/0 1/0 1/0	nonsynon
4	98104052	Inadl	A	C	E478D	exon12	TP	0.074/ benign	1/0 1/0 1/0	nonsynon
5	21545383	Reln	C	A	G805V	exon19	TP	0.995/ prob	1/0 1/1 1/1	nonsynon
5	31566037	ift172	C	A	M1006I	exon28	TP	0.984/prob	0/1 0/1 0/1	nonsynon
5	134692920	Gtf2ird2	T	C	F717L	exon16	TP	0.955/prob	1/0 1/0 1/0	nonsynon
6	36473946	Chrm2	A	G	N246S	exon1	TP	0.01/ benign	0/1 0/1 0/1	nonsynon
6	70093854	Igkv8-28	A	T	V13E	exon1	TP	0.465/poss	0/1 0/1 0/1	nonsynon
6	126924519	D6Wsu163e	A	G	M505V	exon13	TP	0.539/poss	0/1 0/1 0/1	nonsynon
9	21861790	Zfp653	T	C	M466V	exon6	TP	0.788/poss	1/0 1/0 1/0	nonsynon
9	25411078	Eepd1	G	A	C532Y	exon8	TP	0.991/prob	1/0 1/0 1/0	nonsynon
11	50021016	Sqstm1	A	G	C142R	exon3	TP	0.809/prob	1/1 0/1 0/1	nonsynon
11	82883777	Slfn2	T	C	V360A	exon2	TP	0.44/benign	0/1 0/1 0/1	nonsynon
11	95246346	Slc35b1	T	C	V8A	exon1	TP	0.003/benign	0/1 0/1 0/1	nonsynon
12	25742570	Kidins220	A	G	N1688S	exon34	TP	0.967/prob	1/0 1/0 1/0	nonsynon
12	31571934	Fam150b	A	G	M94V	exon2	TP	0.137/benign	0/1 0/1 0/1	nonsynon
12	71317593	Pygl	T	A	Y71F	exon4	TP	0.998/prob	1/0 1/0 1/0	nonsynon
12	77688461	Spnb1	A	T	F533L	exon16	TP	0.001/benign	0/1 0/1 0/1	nonsynon
13	84544914	Gm17618	A	G	Y21C	exon1	TP	unknown	1/11/0 1/0	nonsynon
13	114000186	Esm1	A	G	T46A	exon1	TP	0.587/poss	1/1 0/1 1/1	nonsynon
14	35542591	Wapal	A	G	T799A	exon9	TP	0.811/poss	0/1 0/1 0/1	nonsynon
16	49759470	Ift57	T	A	W169R	exon5	TP	0.473/benign	0/1 0/1 0/1	nonsynon
8	123272248	Irf8	T	C	$601 + 2T > C$	exon6	TP		0/1 0/1 0/1	splicing
4	3710143	Lyn	A	G	T410A	exon12	TP	0.942/prob	1/1 1/1 1/1	nonsynon
4	66590107	Tlr4	A	G	T146A	exon4	TP	unknown	1/1 1/1 1/1	nonsynon
