# Supplementary material for: Unlocking the Bottleneck in Forward Genetics Using Whole-Genome Sequencing and Identity by Descent to Isolate Causative Mutations
Source: PLoS Genet. 2013 Jan 31;9(1):e1003219. doi: 10.1371/journal.pgen.1003219 (PMC3561070; doi:10.1371/journal.pgen.1003219)
Supplement: Table S2 — Variants in IBD Regions with Inconsistent Genotypes. 4 protein sense changing variants within the IBD regions were rejected as causative because they were absent in one or more affected animal, despite good individual depth of coverage (threshold good quality reads at the locus for the inconsistently genotyped animal). (PDF) [file pgen.1003219.s006.pdf]

Chr	Pos	Gene	Ref	Sub	AA sub	Exon	genotypes 1 2 3	type
2	85240307	Olfr992	T	C	N128D	exon1	0/0 0/1 0/0	nonsynonymous
2	155580104	Procr	G	A	A152T	exon3	1/0 1/0 0/0	nonsynonymous
9	40800682	Crtam	A	T	S101R	exon3	0/1 0/0 0/1	nonsynonymous
16	87366449	N6amt1	G	T	R154S	exon5	0/1 0/0 0/1	nonsynonymous
