# Supplementary material for: Unlocking the Bottleneck in Forward Genetics Using Whole-Genome Sequencing and Identity by Descent to Isolate Causative Mutations
Source: PLoS Genet. 2013 Jan 31;9(1):e1003219. doi: 10.1371/journal.pgen.1003219 (PMC3561070; doi:10.1371/journal.pgen.1003219)
Supplement: Table S3 — Forward and Reverse Primers used to Validate Candidate ENU16CH17a Variants. (PDF) [file pgen.1003219.s007.pdf]

Gene	Internal Forward	Internal Reverse	External Forward	External Reverse
Capn3	TCCTCTCTCAACCTCTCAGGA	TCTGAAGCTTATCGGACTCCA	TTCCTCAGCCTGTGTGAGTG	TCGTTTACAGACACCGTCCA
Gzf1	GCTGGAGTCTGTCCTTTTGG	CCCATTGCCCAGTCTCTCTA	AGAAGACCGTGTGCAGCAG	TTGGATGGTGTTTCTGGTGA
Cst3	TCAGCCCTTAGGCATTTTTG	ATCATCCCGGAGTGGGTATT	AGAGCCGGAGCACCCTTC	GGGAGAAGGGTTTAGCTTCC
Wfdc10	CAAGCAAATAACATCTGCTGCT	TAGTCAGAGGGAGGGGTGAC	ATTGTGAAGCCCACCAAGAC	GGACCCACTCTCTGGGTAGA
Frem21	GGTAGCCAGACACAGGTCCA	TTGCTTCTAGGCAACCAAGG	AAAGAAATGTCTTATAATTTCCCCATA	GGATTTTCCAGTCATTTTTCTGA
Inadl	CCACCCCCAACAGGACTA	CTGGTTTCGGGGATCCTG	AGTGAAGTCAGCCCATTTGC	TCCTCTGCTTCCACACTCAA
Reln	TTACGGAATCACTGCTGCTG	GGGAGCAAGTCTGTGCTGA	GGCCCTCCATGCTACTGTAA	TCGTGTCTAGGTTTCTTCAGTTT
ift172	AGGCGAATCATGTCGTCATAC	AGGTGAGTCTCCGGGGAAG	GTGTGTCGCTGAGGAGGTCT	GCAAGTACCGAGAGGCTGAG
Gtf2ird2	TGGCAGCCTACTGTACCACA	CGTCGTCATGTCCACTAGGA	TGGTAGACTCGGTGAACTGG	GGGAGGCATCCAGTGTATTG
Chrm2	TGCAAGGAAGAATTGTAAAGC	CTGAACGCAGTTTTCAGTCC	ACCCGGTGTCTCCGAGTCTA	TCTTTCTCCTCCCCCTGAAC
Igkv8-28	TGGTACCAGGCCAAGTAGTTC	TGATGACACAGTCTCCATCCTC	GCCCCGTAGATCAACAGTTT	AGGTACCTGTGGGGACATTG
D6Wsu163e			TTTGGTATGGAAGGCCAGAG	GTTTGGACTGGGATCTGCTC
Zfp653	CAGCTGGACCCAACAACAG	ACAGGCCCCTTCTCTCCAG	CCACTGACACCTGGTAGAGC	TTATAGCCCACTGGGGACTG
Eepd1	GCTCACAAACCCTTGGATTC	GCTCCAGTCCTTTTCCATGT	CACTGGGCTGTTGTGAGAGA	TTCCTGGGGACTTCCTTCTT
Sqstm1	AAAGGGGTTGGGAAAGATGA	CACCCCAATGTGATCTGTGA	CCCGGCTCACATCAGAGA	TCCCTGCAGAGAAGAAGGAG
Slfn2	GCGCTCAGAGTGGAGAGATT	ACCCATTCCTCTGTGGTAAAC	TCATCGAAGTGCACAAATCC	GGGCATTCATCTGGAGTTTC
Slc35b1	CGGTACCGCTACCTGCTCT	CCTGCAGGATCCCATAGTAGA	GTCGCACGCAGCTCCTAT	CCTCAAGGGTCTGCTCACAT
Kidins220	CACCAACAGAGCCAATCAGA	GCTATCTGAGGAGGCTGCAT	CCCAGCACTGTGACTCTGAA	GGATTTTCCAGTCATTTTTCTGA
Fam150b	CGATTTCTCACCCCTCGTAG	GGACCTGGAACACAGAGGAA	GTTCTCCTGTTTTATCCAGTGC	AAGTGCTTGCTGCACTTCG
Pygl	AATGCCAGCGAAATCAGTG	GCTTCCATGCCTTTGAAGAG	AAGAGCTCACGAAGGCTCAG	TGCTAAGTTCGCTGGTTCTG
Spnb1	TAGCCCTCCATCTGCACAGT	ACCACAAAGACGAGCAGGAG	CACCTGTTAGAGGCCTTCTTG	AGGCACGCTCCTCTCTTGT
Gm17618	TTGCTTTCCTTCTACCTGATGA	TTGGAAAGGAGAGGCAGAGA	TTCCTCACTTTTTATTGCTACTTTT	GCACTTCTCTCAAAAATAGTTAGATG
Esm1	TCTTGCTGCTGACCACACTC	TCCATGCCTGAGACTGTACG	CAGCTGCGAGACATGAAGAG	CTCGTCACCAAAATCATCCTC
Wapal	TGGAACAAGATGCCTCTTCAG	CCAAACCCAACCCAAACTTA	CATGATTCGGCTTTTGGAGT	GCAAAGACTTAAAGGAAGTTTTTATC
Ift57	AAATCCCATGGTTTTCTAGGG	ACCTCACTTAGCTGGGCTTG	TTTGTGGATTGGGTTGAAGAG	AAATCCTGCAGAGCATTCTTAAAT
Irf8	ACCAGCAGTGTCAGCCTCTC	GGACCTCATGACCCCATCTA		
Lyn	AACTCTGAGTAGGAGCCCACA	GGGAATCTTCCCATAGGTGAC	TTTTGATTGTATCTTTTCTATTCCAA	TCACAATGGAGTGGGGTGTA
Tlr4	TGCACATGAATTGTGTCTTCA	GAGATGGAGCGGCAGTTAAG	TTTTATTTTATACAATGATGGTTTCCA	TGGCAAAGGACAGCAATTTT
