# Supplementary material for: Unlocking the Bottleneck in Forward Genetics Using Whole-Genome Sequencing and Identity by Descent to Isolate Causative Mutations
Source: PLoS Genet. 2013 Jan 31;9(1):e1003219. doi: 10.1371/journal.pgen.1003219 (PMC3561070; doi:10.1371/journal.pgen.1003219)
Supplement: Table S4 — IBD Variants in the Second Pedigree Sequenced at Low Coverage (4× per individual) used to examine the false discovery rate at low coverage. The variants in this low coverage pedigree were a randomly selected subset of the filtered shared IBD variants and included both coding and non-coding mutations. Sanger sequencing results: True Positive (TP), False Positive (FP) Failed Sequencing (Unknown - assumed FP). (PDF) [file pgen.1003219.s008.pdf]

Chr	Pos	Ref	SNP	Left (Forward) Primer	Right (Reverse) Primer	Type of mutation	Sanger:
chr10	79354457	C	T	GTGCTGCACACTGCATGG	CGGGTGGGCACTACACTCT	Coding Homozygous	TP
chr8	96929639	A	T	AAATAAACTGGAGAAATCTGAGGCT	AGATGATTTTCCCTCTGGATTTTAC	Coding Homozygous	TP
chr8	81880266	A	G	AGAGTTGGGGTAGCTGCTGTT	TCTTATTTGTCTCTCTCGCTCTCTC	Coding Homozygous	TP
chr10	94306815	G	C	GCATGCATGGAAACTCACAT	GACTGTGGAACGCTGCAATA	Splicing Heterozygous	TP
chr10	77454832	G	C	CAGGAGCTACTGACCTCTTGGT	AAGTGTAGGGCTCTGTCCTCATAAT	Splicing Heterozygous	TP
chr10	62480787	T	A	GTTTTGAAACCTCATAGGTACTGGA	AATAGGAGGAAGAGGGGGTTATTAT	Splicing Heterozygous	TP
chr10	62532691	T	A	ATGAAGAAGATGGCTGAACTACTGT	CCCTTCTAAGATGAAAAACAAGGTT	Non-coding Homozygous	TP
chr8	79028363	A	T	TGAACAAGTAAGGGTTCTTTTTCCT	TATTGACCCCTGCAAGATATAGTGT	Non-coding Homozygous	TP
chr8	68170219	A	T	TGGTGACCAAACTGACAACA	CGATGTGTGTGACAAGCAAA	Non-coding Homozygous	TP
chr10	62533295	T	A	TTCCTACAACTTGTCCTCTGACTTC	CCTGGGTTGTACCCTTTTTATCTAT	Non-coding Homozygous	Unknown
chr10	74458031	A	T	CTCTCACCTCTCCCTCCAGAC	TTTGCAAAAGTGTGCTCTGTTC	Non-coding Homozygous	Unknown
chr8	73517737	G	C	ATTGGACACATTTATAATTCGAGGA	ACTAATATGTCTACTCTGGCGCAAC	Non-coding Homozygous	TP
chr8	94762440	A	C	TATGACTTTCATCAGTGCATTCCTA	CTCTGTGACATCACTGACAATCTTT	Non-coding Homozygous	TP
chr8	75810400	A	T	TTGCTTGAGCATATAAGAAAGACCT	CAGTGTTACAGAAAACCCTGTGGT	Non-coding Homozygous	TP
chr10	100282238	C	A	CAACAGACTGTGTTATTTGTTTATTTATGA	AGAAAATGTAATCATTGGTCCTCAA	Non-coding Homozygous	TP
chr10	75736529	A	T	AAGTAGATGTCTTTACCTTGTTGCG	GCCTATGTACTGAGATGAAACCTTG	Non-coding Homozygous	Unknown
chr8	78014812	A	T	TAACTTATCAGTCGTGGAACTCCTT	TGTCCTCTGGGATTACCACTATTTA	Non-coding Homozygous	TP
chr10	99465763	C	T	CCTGGACATTAAGAATTGTAGACCT	CAGGATCTTTCCATCTTCTATTTGA	Non-coding Homozygous	TP
chr11	53008786	A	G	CAGAGACCCACAGTCAAACATTAG	CCACAAGAAGACCAAGCTATAAAAC	Non-coding Homozygous	TP
chr10	74458034	A	T	TGGCTGTGCTGGTGACTG	GCAAAAGTGTGCTCTGTTCG	Non-coding Homozygous	TP
chr8	87468436	G	A	CAGGCATTGTTAAAGTGGTTAGTTA	GTGTGTCTTGGTCCTCGGAA	Non-coding Homozygous	TP
chr8	91439253	A	G	TGCTGCTGTGAGTTGTAATGTAAGT	GACTCAGAGAAGACGAGAACAGC	Non-coding Homozygous	TP
chr8	96148228	A	G	GAGCTTTTATCGTGAATAGCAACAT	GCTTCTGATTTTTATCCAACAAAGA	Non-coding Homozygous	TP
chr8	77663410	C	T	CTCCCAAGTGCCAGGATTAAAG	GGAAGAGGAAGAAGAAGAAGAAGAA	Non-coding Homozygous	Unknown
chr8	96929639	A	T	AAATAAACTGGAGAAATCTGAGGCT	AGATGATTTTCCCTCTGGATTTTAC	Non-coding Homozygous	TP
chr8	91637313	A	T	ACCTTTCCTAATTCAAAATCAGCTT	AGCAAGGGAGAGATGGTAGAATGTA	Non-coding Homozygous	TP
chr8	72409696	A	G	CTTGCTAGATGCGGTCCTAATTAAC	GAGCTGTGCCCAGATTGTAGTAG	Non-coding Homozygous	TP
chr10	75494347	C	A	ACAGGCAAGAAGGAAAAGAATGAG	ATTCGAATTTCAGTTTTCAGTTTTG	Non-coding Homozygous	TP
chr8	70491385	A	T	AAAAAGGAAATACATCCGGTATAGG	TGACTGTTTGTGCCATCTTTACTTA	Non-coding Homozygous	TP
chr8	84771825	A	G	ATCCATGTGCAATTTAATTCTCTGT	AACACCTTTTTGCTTATTTTCACAG	Non-coding Homozygous	TP
chr10	74503812	G	T	TCTGTGCGAAAGCTGGTAGA	GGCCCAAGCTCACAGTAAAA	Non-coding Homozygous	TP
chr5	64196341	A	G	ACGAAAGCATAGAGCAGTTATTCTG	AGCCTTGCAATATACAGGTAAGGTA	Coding Heterozygous	TP
chr4	123152578	T	C	GGATACAAACTGACCTTAAGTTATTGA	TCTGGAATCCCTCTGGTCAT	Coding Heterozygous	TP
chr8	112051551	A	T	GTCTCTTCTTTGACCTCCTCAGTT	GCAGTCTATTTTGGATGTGCTTT	Coding Heterozygous	TP
chr5	38711842	T	A	AGCCTGCGAGCATCCATC	TGATGGGGTTGAGAGAGGAG	Coding Heterozygous	TP
chr10	57929942	C	T	TATTTTTGCAAACTAAGGCATTTTC	GAATATTTGGTAGGAGATGGTGTTG	Coding Heterozygous	TP
chr10	79354457	C	T	GTGCTGCACACTGCATGG	CGGGTGAGGCACTACACTCT	Coding Heterozygous	TP
chr10	121229763	A	G	GAGGTCAGGAGCTAACAATCTCAC	AGACCTACAAATTAAGGACAGCCAC	Coding Heterozygous	TP
chr4	118399428	G	T	CTTTGCAATGAATCTTCCTTACTGT	CAGATCCTTAGAATGGCTATGAAGA	Coding Heterozygous	TP

chr11	65831008	A	G	TAGAGTTCATATATTTCCTTGGGGC	TAGCTCTTCTCTCTCTCTTTCTCCC	Coding Heterozygous	TP
chr4	102102720	C	G	GTGTCTAGGTTGGTGATTTACAAGG	TAGTGCTAGAAGCAGTCTAAATGCC	Coding Heterozygous	TP
chr4	147129703	T	C	CTGATGAACGCTAAGATGAGATTTT	CAGATCCATCTTAGTATCCATCAGG	Coding Heterozygous	Unknown
chr11	33969900	T	C	CTCTGACTCTCTGTTGGGTACTGAT	AGCATTAAGATCAATGTAAAGCCAC	Coding Heterozygous	TP
chr5	145582831	A	T	AGTCCTGAACTTCTTGCTAACTGTG	AAAATGTAACTAAGGACTACGGGCT	Coding Heterozygous	TP
chr4	102915369	T	A	TGGGCATATATTTCCTTGATGTTTA	CTGTTTAGACAGAATACGCCAATC	Coding Heterozygous	TP
chr5	77830595	A	G	AACCAATTCCCAAACTAGGTAGACT	TAATACAGTTCCTCATGTTGTGGTG	Coding Heterozygous	TP
chr4	102102719	G	A	GTGTCTAGGTTGGTGATTTACAAGG	TAGTGCTAGAAGCAGTCTAAATGCC	Coding Heterozygous	TP
chr8	126957907	G	T	GGTCTCGTCCTCCTCGAGTT	GCTACGAGAGCCTGACACATC	Coding Heterozygous	TP
chr11	59598709	T	C	ACATACTCGGTGTCCTCCATAATC	TCTCTCTGTAATCTCAGTACCACCC	Coding Heterozygous	TP
chr6	55013134	G	A	AGATTTTGATCTTGTTTTTCCTTCC	CTCCCTGAGAACCTTACCTGTTC	Coding Heterozygous	TP
chr6	83002774	G	A	TGTAAGACAGGCTCACTTTTCTTTT	TCCTGGTATACAGACAGACAGACAG	Coding Heterozygous	TP
chr6	113434047	G	C	GTGCTTGAGCCTGTTTCTGAG	CTTACCCAAATGCCATACAGTTAAA	Coding Heterozygous	TP
chr10	93596769	T	C	ATAGAAGCCTACCGAAAAGATGTG	GAATTCACTTGTGCAAATCATACAC	Coding Heterozygous	TP
chr4	143401586	A	G	GGAATGTAGGAAGATGAGAAATCAC	TAACCAAAGCCACAGTCATAGAAAT	Coding Heterozygous	TP
chr11	94967924	G	T	AGCGTGTTTAGAGGAAGAAGAGAG	GCTCCTATCCTTTCCCTCTGTAG	Non-Coding Heterozygous	TP
chr2	43148044	A	G	AAAAGTGTTGGGTTTGGTGTCT	ACTTTAGCCTTAATGCTCAGAGAAG	Non-Coding Heterozygous	TP
chr4	119876618	T	C	TATCACACTGGACTTTCCTCTTTTC	ATGACTGCTGTCCCCTTGTTAC	Non-Coding Heterozygous	FP
chr6	69167249	A	T	TCTCTCTGACTCTCTCTCTCTCTGG	GGATACCATGGGAAATATAAATGAA	Non-Coding Heterozygous	TP
chr19	29483659	A	T	GGAACAAAGAAAGAGCTTATGAGTG	TTTTTCCACCTTATATTGCATTTTC	Non-Coding Heterozygous	TP
chr10	108727258	T	A	ACACAATCTGTCAAATAGTTCTTGG	TCGTTTACAGAAACTAGGTGTTTGC	Non-Coding Heterozygous	TP
chr9	31588417	T	G	GCATTCCAAATCCTAGATGACTATG	GTTTCTTTGGGGTTACTGGTTAGTT	Non-Coding Heterozygous	TP
chr10	65179218	A	G	TAAGATTAGTTGGGCTCTTCTGCTA	AAATTGCAGTTAATGTTGTTGGAAT	Non-Coding Heterozygous	TP
chr5	150645401	C	T	ACCCTGAAGCTACTGAACATACATC	CTGAAGACAGAACATCAGAAGTTGA	Non-Coding Heterozygous	TP
chr2	17426021	A	T	ATTTTAAATGTCTCAGATGCAGGAG	GACTGCCTTGTCTATTTATGAGGTG	Non-Coding Heterozygous	TP
chr6	100774589	A	G	AGTGAGAGACCTCGTCTCAAGG	TTCTCAACTCTCAAGGGACATAAAC	Non-Coding Heterozygous	TP
chr4	145462750	C	G	TCTTTCTCTCCCTTAAGTAGCTTCC	CAAGCCTTTAGGATTTGCTTTAATC	Non-Coding Heterozygous	FP
chr2	28619987	G	A	ACTAGGCCCCATCTTCTAGAGTTT	GAGGTAGAGACAGGAGGATCAGAAT	Non-Coding Heterozygous	TP
chr6	72034052	A	T	AAGAAGAAGAAGAAGAAGAAGCAGC	TCCCAAGATAGAGAGAGAGAGAGTG	Non-Coding Heterozygous	TP
chr6	44456662	A	G	TTCAGCCCATTATCCTTGGT	TTCCTTTCCCCACCTATCCT	Non-Coding Heterozygous	TP
chr2	31644666	A	T	CATCTCCATAGTCTTTTAGAACCCA	CTAGCTGCAAGAACAAGGCTCT	Non-Coding Heterozygous	TP
chr10	113805663	G	C	AAATCTCGTGGGGAAGGACT	AAGCAAGCAAGCAAGGAAAA	Non-Coding Heterozygous	FP
chr11	6605800	C	T	TTTCTTAATAGACATGTCCCCTGAC	GAGGTGGACCTAATGATCAATGAC	Non-Coding Heterozygous	FP
chr19	39996078	A	G	TGCACATACTAGTCTCAGCAGAATC	GCAACAGTAGGTGAGAATGGTAATC	Non-Coding Heterozygous	TP
chr8	32391933	A	T	AATTGCTAGTGCTACTGTGGTAAGG	CCCCATGATCAACATTTGTAAAGTA	Non-Coding Heterozygous	TP
chr6	100153871	A	G	CTCCCTCATTCATGCTGTCTTAG	GAGACCAGATTAGAATGGAGACAAA	Non-Coding Heterozygous	TP
chr11	28123635	T	A	TCACTGTCTTTGTATTGAGTTAAGGAA	TAAAACTGTGATTCTGTGATCCTGT	Non-Coding Heterozygous	TP
chr4	124138040	C	G	GTGTGTGTGTGTGTGTGTGTGTGTA	CACATACTCCTTCCATCATTTCTTT	Non-Coding Heterozygous	Unknown
chr10	66292950	G	A	AGGTAGGTAGGTAGGTAGGTAGGTAGA	ATCTGTCTGTCTGTCTGTCTGTCTG	Non-Coding Heterozygous	Unknown
chr10	67397252	A	T	CACCTGTACATGCACATACATCTTA	ACATCCAGGAAGTGTATGAATAAGC	Non-Coding Heterozygous	TP
chr8	3832950	A	G	AAAGGACAGTCTTAAGCCAGATACA	AGGGGAGACTACATTTCCTCTCTT	Non-Coding Heterozygous	Unknown

chr11	20336828	T	A	ATATTTCCAATATTCTTCCTCGACC	TGTTGATTGGTAGTATGAACAGGAA	Non-Coding	Heterozygous	TP
chr11	5265846	T	A	AAAGTTGCTTATGCCCCAATTAAAG	TGGGGTAAGAACTAATCCTATGTGA	Non-Coding	Heterozygous	TP
chr4	124954916	G	T	AGTTCCTGGTACTTTTTGGAGAGAT	CTCAACACAAGCAACTTAAGAGAGG	Non-Coding	Heterozygous	TP
chr2	12336455	A	G	CACATTAAGACAATCAAATGTCAGG	GGCTAACTTGGTGTTTATTACATGG	Non-Coding	Heterozygous	TP
