# Supplementary material for: Unlocking the Bottleneck in Forward Genetics Using Whole-Genome Sequencing and Identity by Descent to Isolate Causative Mutations
Source: PLoS Genet. 2013 Jan 31;9(1):e1003219. doi: 10.1371/journal.pgen.1003219 (PMC3561070; doi:10.1371/journal.pgen.1003219)
Supplement: Table S5 — Non-IBD Variants in the Second Pedigree Low Coverage. Non-IBD variants selected randomly from the low coverage pedigree (4× coverage per mouse), showing SNP genotype from variant caller for each mouse. Sanger sequencing results: True Positive (TP), False Positive (FP) Failed Sequencing (Unknown - assumed FP). (PDF) [file pgen.1003219.s009.pdf]

Chr	Pos	Ref	SNP	Left (Forward) Primer	Right (Reverse) Primer	Mouse1	Mouse2	Mouse3	Sanger:
chr10	51889817	A	T	TGAAGCTTTGAATAATTTAAGGACAA	GAATAGTGACTGAAATGCTGTTTCC	1/1	0/1	0/1	TP
chr1	46261086	A	C	ATGAAATACAGTGAAAACATGAGCA	CACTACTGTAGGAGTGCTGACACAT	1/0	1/1	1/1	FP
chr11	72731358	T	A	GATGTTGTCTGAAGAAAGGGTTGTA	TACCTTTATGACCTTTGAATGAAGC	1/0	1/0	1/1	TP
chrX	3907117	G	A	TCAGAATCTGTAGTAGAGAAACCCA	GAGGTCCATGAACATTAGAACTTGT	1/1	1/1	0/1	FP
chr11	101187980	T	C	CTATATCAACACAGCCTTCATGAGT	CTTTCTGAGCTCACTCCTTGATTT	0/1	0/1	0/1	TP
chr7	149044528	T	C	ACTCCTCTCACAGTCACCTCAGT	GACAAGGAAGCCTTTGAGGTT	0/1	0/1	0/1	TP
chr12	114018097	C	G	ATCTTGAGGCTGGGCATCT	CTGAAGACCCCTGACCTGAG	1/0	0/0	1/0	TP
chr14	43347611	G	A	CTTGGTCTGGTGTCTGTGTGTATT	GAGAACCCTAGAATCTTCTGTGATG	1/1	1/1	1/1	FP
chr2	145747631	A	C	ATACATTCAACTTGGGATCTCACC	AAAGGATACATAGAACTGGAACTGC	0/0	1/0	1/1	TP
chr2	82823507	G	T	AAAAAGTAAACGATTCCACAGAAGA	AAAGGAATTTGCCTTGACATAAAA	0/1	1/1	1/1	Unknown
chr5	139048775	A	T	ATACTGCTTTTGTGTTGAAGGATTT	GTTTATTCTGTGGAAGGACGTGTAT	0/0	0/1	1/1	TP
chr11	68628466	G	T	GTGGAGAGTGAGCCTTGTTTAGA	TTGACATCACTGGTCTTACTTTCTG	0/1	0/1	./.	Unknown
chr4	137104386	C	G	AATATGTGTGCAGCGTTCTGAG	CAGGTCTCAAAGAGCTTTTCTTCT	0/1	1/1	1/1	TP
chr14	66378562	A	T	GTGACATGGATTTCACAATGATTC	GTACCTTACCTCACCATCCTTACCT	0/0	0/0	1/1	FP
chr6	126688537	G	A	GATGTGGAGTCGGAAGGTAGC	ATGACCACGGTAGGTTATGGG	0/0	0/1	1/1	TP
chr11	70028825	C	T	AGAAGAGGGGCTAAAGAATGTTTG	AAAGTATCAAGGAGGAACTGCAAG	0/1	0/1	1/1	TP
chr6	32135423	A	T	TCTTATCAATGAGGTCAGCCAGTAG	AAGGTGTTCCTGCTCTCTTTCAT	0/1	0/0	0/1	FP
chr1	53549974	T	C	ATCTGGTGTAATTCTGATAGGCTTG	GAATGAAGATTTTCAACTCAAAGGA	1/1	1/1	1/1	FP
chr2	83262425	A	T	AACTGTGAAAGTCTTGGTTTAGGTG	ATCTCAGAGAATAGAATGTGAAGCC	0/0	1/1	1/1	FP
chr9	21356924	T	A	AGGTCGTAAGCTGAGGTAAAAGAAT	AGTTCAAAAAGCTACTCTATGGCAA	1/1	1/0	1/0	FP
chr4	43597914	C	T	AAAAGCACCCCAAAGATAAACTTC	CTCCTCCAGAGTCAGCAGAAAT	1/1	1/1	1/0	FP
chr4	3182839	T	C	TATTGGCTCATAATATTGCTGTTGA	TCCTCTCGAGGACTTTGAGTTATTA	1/0	1/0	1/0	Unknown
chr5	87716559	C	T	CAGCTATCATTCACTTCAATCAGAA	GGCCTGGTTGTTAATGGTAGATAAT	0/0	0/0	1/0	FP
chr1	4329977	G	A	GAAAATTTCCTGAATAGAACAGCAA	CATTTGATTTTTGATTTTCTGGAGT	0/0	0/0	1/1	FP
chr10	3042685	T	C	TTGTATTTACAACTATAGGCCAGGTG	CACACACACAGAGAGAGAGAGAGAG	0/1	0/0	0/1	FP
chr2	94795429	A	G	AAACAGGTTTTTCAGAGTTGATGAC	TGAAGTTATAATCATTCACAGGGGT	0/0	1/1	1/1	FP
chr3	82193049	C	T	GGATAGTACCATGACTTATGTCCCA	ACAGAAACGTGGATTAAAGTACAGG	0/0	1/0	1/0	TP
chr3	91370835	A	G	ATAGCTGACTGTGAGCATCCACTAC	CATGGAAGGAGTTAAAGAGACAAAA	0/0	0/0	0/1	FP
chr4	89184839	A	G	AGAATGGTTTCCAAGCTACAAGAT	TGTTCAAACATATTCCAGTAAGCAA	1/0	1/1	1/0	TP
chr2	165946667	G	A	GGTGGAGTACTAAGCATTGGAAAAG	ACAATCTCACATGCTAGATCAACAA	0/0	0/0	1/0	FP
chr6	136724323	A	T	TAAAATGTCAAGAAAACACATCCTG	AGCTTACAACTCTCGGAAACTCC	0/0	1/0	1/1	TP
chr14	66527259	C	A	AGGGACGAATACATCTTTCTAGGAT	AAAGAAAAGAGCTGAATGTTGAGAA	0/0	0/0	1/1	FP
chr16	18592638	T	C	CTCTCCTCTCCTCTCCTCTCCT	AGGATCTCTGCATTGGAAAGTTAG	1/1	./.	./.	Unknown
chr5	143163864	A	T	GCTGTAATTTTGCTCCTGTTATGAT	CTTTCAATGTGCAAGAGGTGAAG	0/0	1/0	1/1	Unknown
chr1	13025454	A	T	TCAGAAATGAAAAGGGAACAATAAA	TCAAAATAGCAAAACCTTTTTGAAG	0/1	0/1	0/1	TP
chr1	6170534	G	T	CACCTGAGATCATTGGAAAACATA	TCTTTTTACCTTTCCTGAACTTCG	0/0	./.	1/1	TP
chr3	74125312	G	T	GAGTGTGGGCTTAGTCCTATAACAA	TGGAAAATGAACCAAAATAGTCTCT	0/0	0/1	1/1	TP
chr11	99020131	A	G	CCGAATTCTTTCTCTCCTACATTTC	GTCCAAACACTAAAGATCCTTGTCC	0/1	0/1	0/1	TP
chr3	83183859	C	T	GAGTTACAGAGACAAAATTTGGAGC	CTCCTTGGGTACTTTCTCTAGCTCT	0/0	1/0	0/0	FP

chr2	102124138	A	G	CCCTAGCCCCATTTATTTATGTTAT	TAAAGCATGAACGTAAACATTGAAA	0/0	1/1	1/1	TP
chr6	33734748	A	T	ATAAGAAGTAGAAAGAGTGCGCTTG	GGACTTCACTGGAGACATTTTACAT	1/1	0/0	1/1	TP
chr17	84861317	G	A	ACACACACACTTACTGCCCTAGTCT	ACTAGGACAGTAAGTGTGTGTGTGG	1/1	./.	1/1	Unknown
chr16	63177786	A	G	CACTCCGTGAAACAACATACAATAG	GTAGAAGAGAAAGGAAGACCTGGTT	0/0	0/1	0/1	TP
chr10	53641804	T	A	CATTCTCATTTAATGCAACTGAACA	CATAGTTTATTTAGGAGGCAGCAA	1/1	0/1	0/1	TP
chr6	60489599	G	T	GAATCTCACTTAGAAGGGGGAATAA	TCACTCCTATTGATTCTTAGATGCC	0/1	0/1	0/1	TP
chr3	59781263	A	G	GACTTACACACACACACACACAAAA	GACTAAGACATTTGGGAAACTGAGA	0/0	1/0	1/1	TP
chr11	78281164	T	A	AAAAAGCTAAAATTCCTGCTGC	GTGTCACTACCGAGTTAGCTGAGTT	0/0	1/0	1/1	TP
chr6	122418894	A	T	TGGATGTGTCTATATTACGAGATGG	ACGGTATCTTTACCTTTGTGTCTTG	0/0	1/0	1/1	TP
chr11	81178333	C	A	AGTTTTAGCAAGTGAAAGTTGGAGA	TAAGTGGATATCCTACCCACCTGTC	0/1	0/1	1/1	TP
chr6	133915812	T	A	TGTATTGGGTTTTATGTGAGGTCTT	TAGACATAGCCATTTTACCAAAAGC	0/0	1/0	1/1	TP
chr6	38167512	A	T	CTCCCCCAGTCAGAGACTAGTTAAT	AGACTCGCTCATCTTACTGACCTAA	1/0	1/0	1/0	TP
chr17	38389993	T	C	TTTCTTTCTCAGCCTGTTTATCAAT	CTCTCTCTCTCTCTCTCTCTCTCCC	0/0	1/0	1/0	Unknown
chr2	115767708	T	A	CTTTTCTCGCTGAAGTCATCTAAAG	GGAGAGAAAGTGCAACTCATTTTTA	0/0	1/1	1/1	TP
chr6	34942306	A	G	ATTCAGCTGTATACATTTTTGAGGC	CGTTCTTAACTCTTCACAGTCATTG	1/0	0/0	1/0	FP
chr3	56214691	A	G	AAACCTTTAGTGTAGATGTAGGCCA	ATCAAAACAACTTTAGTGGCAAGTC	0/1	./.	0/1	FP
chrX	84319042	T	C	CTCTTTGATGATTTGTTTTTGCTTT	TCTTTAATCTCATCACTCAGAAGGC	./.	1/1	1/1	Unknown
chr2	82823507	G	T	AAAAAGTAAACGATTCCACAGAAGA	AAAGGAATTTGCCTTGACATAAAA	0/1	1/1	1/1	FP
chr2	25471530	G	A	CAACAGACTTACACTGACGCTCTT	GGACTCAGCCAAATAGGCTTC	1/0	1/1	1/0	TP
chr10	59791020	T	C	TTATATTTAGGGAAATGGAGGGAAG	ATACATTTCACAGAAGTGCTGAAGG	1/1	1/1	1/1	TP
chr14	44082649	G	A	GAACTGGAATTAGATCTTCGTTGAA	GGCTATACTCAACAAAACTGGAAAA	1/0	1/0	0/0	FP
chr5	144295535	A	T	TATTTATTTTTAATTTAGCCGGGCA	CTAGGGTCCCCAACTCACTATGTA	0/0	0/1	1/1	TP
chr4	137271581	C	A	CATGTAATAGACCAGGACAGGACAG	AATAGTAATTGAAGTGCTCCTGTGG	1/1	1/0	1/0	TP
chrX	4767755	G	C	AGTGGACCTGTGAGTAAAAGCC	GCATCTCTATTCACACTGACAACAC	1/1	./.	1/1	Unknown
chr12	118561783	A	G	GTTTCTTACCACTTCCTCAGACTCA	AGAAGTTCTCAGTTGTGCCTCTCT	1/1	1/0	./.	Unknown
chrX	3918573	T	G	ACTACTTTACCTTGTGCCACTATGC	CAGGAAAATCTCAACAGAACTCAAT	1/0	1/0	1/0	TP
chr6	142116493	T	A	TGTGCTTTCATGTTGCTACTTTATT	AAATGAAAGATTCTCGAATTCCC	1/1	0/1	1/1	Unknown
chr4	105692962	A	T	TTTGGGGCTCCAATAATTAAGTAAG	CTGGTCCACTTGCTTTATCATTAGT	1/0	1/1	1/0	TP
chr19	43900761	T	A	GAGCTCAGTTGTCAGTATGAGTCAG	CTGACATCCTCACACAGACATACAT	1/1	1/1	1/0	TP
chr17	30176494	A	G	TTTGAAGATCATGGATGCATAGTAA	AAAGGAGATTTACAGACGAGTTTCA	0/0	1/0	1/0	TP
chr3	60540665	A	G	GTAATTGTATGCATATTTCTTGCCC	ATGACTACTTGTGGGAATCACTTGT	0/0	0/1	1/1	Unknown
chr14	81758650	A	G	TTCTCAACAATAAAAGAACCTCTGG	AGACAGGGTTTCTCTGTGTAGTCCT	0/0	0/0	1/0	Unknown
chrX	124413795	T	C	ACAGTGAGAAAATAATGAAGCCAAG	ATCTTCTCTGAGACTCTGCTACCTG	1/1	1/1	./.	Unknown
chr1	54858008	A	T	TGTTCATTAAGATTGTTGACAGTGA	AGTAAGGCTGTTGGATATACCTGTG	0/0	1/0	1/1	TP
chr17	84843952	A	T	TGGTTAGAAGCTGCCTAGTAAAAGA	CAGTTTGGCCTTATTTGTAATTTTG	./.	1/1	1/1	TP
chr3	65616079	T	C	ACTTGCTTTTCTCCTTTTACCATTT	CAGGAGTCAAACCAATCATTTCTAT	0/0	0/1	1/1	TP
chr2	118251979	A	G	CAGCATCTGATTGTTATCTTTGGA	AAAGAGAAAAGAAAAACCAACAACA	0/1	1/1	1/1	TP
chr12	5066118	C	T	GAGTCCTTTGGTAGCCTGGTATC	AAGCAAGGCTACCAAAGTACTCC	0/1	0/1	0/1	Unknown
chr14	7559653	T	C	TGAAGAATTATGTTCCTCTTAAGGC	CTTCTCTTCACATGGTGGTCTATCT	1/1	1/1	./.	TP
chr11	107967175	G	A	CCAGACCTCACTTTTCTTTCATTTA	GGACACCTGTCCTCTGGTCC	1/1	1/1	1/1	Unknown
chr6	138241120	A	T	GAGAGAGAGAGAAAGAAAGGAAGGA	TCTTTCTTTCTTTCTTTCTTCCTCC	0/0	0/0	1/1	Unknown

chr5	92151731	A	T	GTCCCTGAACTGTGGTAAGTTAGAA	CATGCTTTACACAGCTTTGACTCTA	0/0	0/0	1/0	FP
chr5	101596050	A	C	TATGTCACAGCAATGAATAAAGCAT	TGAACCTATCTTTAAGCCCTTTTCT	0/0	0/0	1/1	FP
chr5	146494346	A	C	GAAGAATTGAGTTGGAATTTTGATG	CACAAATAATACAAAAGACCTTGGC	1/0	1/0	1/0	TP
chrX	4260792	T	G	AATCACAGGAGACAGAGATAAGCAG	ACATTATCACCAGCTTTCTGTTTTC	1/1	1/0	1/1	TP
chr4	105057916	A	G	TGAGGCTGAAATCTTATCCTTTCTA	TTTTGTAGGGTTGAGCAAAATAATG	1/0	1/1	1/0	TP
chr3	72456852	T	C	GGTACTTTCTCTAGCTCCTCCATTG	TGCATCAAATTGGATAACATCAATA	0/0	0/0	1/1	TP
